# Geochemical compositional controls on DNA strand breaks induced in in vitro cell-free assays by crushed rock powders from the Panasqueira mine area, Portugal

**DOI:** 10.1007/s10653-020-00653-9

**Published:** 2020-07-09

**Authors:** Hatim Badri, David A. Polya, Andrew. C. Povey

**Affiliations:** 1 Department of Earth and Environmental Sciences and Williamson Research Centre for Molecular Environmental Science, University of Manchester, Williamson Building, Oxford Road, Manchester, M13 9PL UK; 2 Department of Environmental Health, College of Public Health and Health Informatics, Umm Al-Qura University, Makkah, Saudi Arabia; 3 Division of Population Health, Health Services Research and Primary Care, School of Health Sciences, Faculty of Medicine, Biology and Health, University of Manchester, Manchester, M13 9PL UK

**Keywords:** Particles, Heavy metals, DNA strand breaks, Cell-free assay, Toxicity, Panasqueira

## Abstract

DNA strand breaks are a common form of DNA damage that can contribute to chromosomal instability or gene mutations. Such strand breaks may be caused by exposure to heavy metals. The aim of this study was to assess the level of DNA strand breaks caused by µm-scale solid particles of known chemical composition with elevated heavy metals/metalloids, notably arsenic, using an in vitro cell-free DNA plasmid scission assay. These samples were incubated with and without H_2_O_2_ to see whether damage occurs directly or indirectly through the Fenton reaction. Levels of DNA damage in the absence of H_2_O_2_ were < 10%, but in the presence of H_2_O_2_, all samples showed higher levels of damage ranging from 10 to 100% suggesting that damage was being incurred through the Fenton reaction. Using bivariate correlation analysis and multiple linear regression, manganese oxide (MnO), sulphur (S), copper (Cu), and zinc (Zn) concentrations in the particulates were found to be the most significant predictors of DNA damage. The mechanism of this DNA damage formation has yet to be thoroughly investigated but is hypothesised to be due to reactive oxygen species formation. Further work is required to assess the extent of contribution of reactive oxygen species to this DNA damage, but this study highlights the potential role of chemistry and/or mineralogy to the extent and/or nature of DNA damage caused by particulates.

## Introduction

Heavy metals and metalloids are natural elements characterised by their high densities, atomic weights, or atomic numbers (Koller and Saleh 2018). Our natural environment contains a large number of heavy metals and metalloids, such as arsenic, cadmium, chromium, and nickel, that become sources of exposure to humans as a result of natural or anthropogenic processes (Alloway 2013; Tchounwou et al. 2012; Bhavani and Sujatha 2014).

It is well established that exposure to many heavy metals and metalloids causes adverse health effects in humans. Many heavy metals and metalloids are classified as human carcinogens by the International Agency for Research on Cancer (IARC 2018). A variety of signalling and cellular regulatory proteins that are involved in important processes such apoptosis, cell cycle regulation, DNA repair, DNA methylation, cell growth, and differentiation are affected by exposure to heavy metals and metalloids (Kim et al. 2015; Engwa et al. 2019). Any disruptions to these processes can lead to cancer (Engwa et al. 2019). The main mechanism of inducing these disruptions is oxidative stress. Certain heavy metals and metalloids, such as arsenic, iron, copper, chromium, cobalt, and vanadium, are known for their ability to produce reactive oxygen species (ROS) such as superoxide ion, hydrogen peroxide, and hydroxyl radical by utilising the Fenton chemistry/Haber–Weiss reaction (Jaishankar et al. 2014; Manoj and Padhy 2013; Szivák et al. 2009). Their production results in oxidative stress, a state where cells have elevated levels of reactive oxygen species (ROS), which causes damage to proteins (e.g. protein fragmentation), lipids (e.g. lipid peroxidation), and DNA (e.g. DNA strand breaks) (Schieber and Chandel 2014; Barrera 2012; Rehman et al. 2018; Engwa et al. 2019).

Given the known effects of exposure to heavy metals and metalloids, this study will focus on the effect of samples with known mineralogical, chemical, and physical characteristics on DNA strand breaks. The samples were collected from inside and around the Panasqueira mine area in Portugal and selected for the wide range of heavy metals/metalloids and major oxide composition. The overall objectives of this study were to (1) determine the level of DNA damage induced in the presence and absence of H_2_O_2_ using the plasmid scission assay and (2) identify the main determinants of DNA damage formation using bivariate correlation analysis and multiple linear regression.

## Materials and methods

Rock samples were collected from in and around the Panasqueira mine, Portugal. After crushing, the resultant crushed rock powders (CRPs) were analysed by means of X-ray diffraction (XRD), X-ray fluorescence (XRF), and particle size analyser to investigate their mineralogical, chemical, and particle size characteristics, respectively. The ability of CRPs to cause DNA damage was investigated using an in vitro cell-free plasmid scission assay. A more detailed description of each method is provided below.

### Sample collection

Whole-rock samples ranging from 0.5 to 1 kg in weight were collected from the Panasqueira mine area in Portugal in February/March 1984. Every sample was broken up with a carbide splitter and reduced to millimetre-sized particles in a jaw crusher. A portion of the crushed material was then placed in a Cr-V stainless steel Tema Mill (TEMA Machinery Ltd., Woodford Halse, Northants, UK) and further crushed to < 50 µm powders. Pressed powder pellets were then prepared by standard techniques as outlined in (Polya 1987, 1988). Throughout the whole process, every piece of equipment was thoroughly cleaned after each sample treatment. Around 250, crushed rock powders (CRPs) were eventually obtained and subsequently stored in sealed individual zip-bags at room temperature. For the purpose of this study, a subset of 24 samples were selected on the basis of their chemical compositional variability, particularly with respect to arsenic, to test their association with toxic effects.

### XRD analysis

Sample preparation involved grinding ~ 0.1 g of crushed rock powder, mixing with ~ 1 ml of amyl acetate, using an agate pestle and mortar, transferring the resultant slurries to a glass microscope slide and air drying. Measurements were carried out on a Bruker D8 Advance diffractometer, equipped with a Göbel Mirror and a Lynxeye detector. The X-ray tube had a copper source, providing CuKα1 X-rays with a wavelength of 1.5406 Å. Samples were scanned from 5–70° to 2*θ*, with a step size of 0.02°–2*θ* and a count time of 0.2 s per step. The resultant XRD patterns were evaluated using EVA version 4, which compares experimental data to standards from the ICDD (International Centre for Diffraction Data) Database.

### Particle size distribution

Particle size analysis was conducted at the British Geological Survey (BGS) in Keyworth by Thomas Walker (Walker, unpublished work). Each sample was weighed out into 2 vials of 0.25 g and suspended into 10 ml solution of Calgon (25% sodium hexametaphosphate). The samples were then shaken and mixed for 30 s using a vortex mixer at 2500 rpm before being analysed using a Beckman Coulter LS 13 320 Particle Sizing Analyser. Each sample was analysed twice. Each resultant particle size distribution was characterised by five parameters [P1 (% 100 µm peak), P2 (% 10 µm peak), and P3 (% < 1 µm), D10, D50,] chosen based on their ability to describe the sample as a whole or describe the largest fraction peak (100 µm), the modal fraction (10 µm), and the nanoparticle fraction (less than one µm). D10 is the diameter at which 10% of a sample’s mass is comprised of smaller particles, while D50 is the diameter at which 50% of a sample’s mass is comprised of smaller particles. Both D10 and D50 values were calculated from the distribution, and statistical analysis was conducted using Gradistat© software on Microsoft Excel (Walker, unpublished).

### Plasmid scission assay

The ability of CRPs to cause DNA strand breaks was investigated using the plasmid scission assay as described previously (Dumax-Vorzet et al. 2015) with minor modifications. When plasmid DNA runs through an agarose gel, three bands are observed. Supercoiled plasmid DNA is the native form (covalently closed circular DNA) where there are no strand breaks. When one DNA strand is cut, the resulting nicked or relaxed plasmid DNA will have a floppy open circle structure. When both strands of the plasmid are cut, the result is linear plasmid DNA. These three forms have different migration speeds where supercoiled plasmid DNA is the fastest as it does not have any strand breaks and its compactness sustains less friction against the agarose gel. Linear plasmid DNA runs through the gel slower than supercoiled plasmid DNA but faster than nicked or relaxed plasmid DNA. Nicked or relaxed plasmid DNA is the slowest due to its large floppy circular nature. In brief, pchAT plasmid DNA (kindly provided by Prof. Geoff Margison, purified from E.coli in lab using Miniprep (Qiagen, The Netherlands)) (5 ng) was diluted to 20 μl in an elution buffer (10 mM Tris–HCl pH 8.5) with different levels of CRPs and H_2_O_2_. Samples were incubated for 1–5 h at 37 °C. The reaction was stopped by adding loading buffer (Promega blue/orange 6 × loading dye) and the whole reaction mixture loaded onto 0.6% TBE-agarose gel. Electrophoresis was conducted at 90–100 V for 45 min–2 h in 1 × TBE buffer. The different forms of plasmid were visualised on a Typhoon 9200 variable mode imager. The intensity of the different forms of plasmid in each lane was analysed using ImageQuantTL (GE Healthcare Life Sciences), and the level of damaged plasmid in each sample was calculated as shown in Eq. (1).

1 $${\text{DP}} \left( \% \right) = \frac{R + L}{R + L + S} \times 100$$

where DP is the percentage of DNA damage, *R* is the relaxed form of plasmid DNA, *L* is the linear form of plasmid DNA, and *S* is the supercoiled form of plasmid DNA.

In each experiment, positive and negative controls were added. The positive control was H_2_O_2_ (3.5 mM), pchAT plasmid DNA (5 ng), and FeSO_4_ (25 µM) in elution buffer. The negative control was H_2_O_2_ (3.5 mM) and pchAT plasmid DNA (5 ng) diluted in elution buffer.

### Statistical analysis

Data obtained from each plasmid scission assay were described using the mean, standard deviation, minimum, and maximum values. A one-way analysis of variance was used to compare each group individually to determine whether the levels of DNA strand breaks varied significantly between samples. A bivariate correlation analysis was then conducted to examine possible associations between DNA strand breaks and the physiochemical composition of the samples. All the significant variables from this analysis were then plotted against the percentage of DNA strand breaks to examine the correlations and entered into a backward stepwise multiple linear regression model. The least significant variable was eliminated step by step, and all the models were compared using Bayesian Information Criterion (BIC) to choose the best explanatory model. Statistical analyses were performed using SPSS Statistics version 22. Graphs and scatterplots were created using Microsoft Excel 2010.

## Results

### XRD analysis

The crystalline minerals identified by XRD in the CRPs were mostly silicates with minor sulphides. These included quartz, muscovite 2M1, dravite tourmaline, and albite (Tables 1, 2 in supplementary material). The most abundant crystalline minerals found were quartz (Modal abundance = 40%, SD = 17%), muscovite 2M1 (*M* = 32%, SD = 19%), and dravite tourmaline (*M* = 11%, SD = 16%) with lesser amounts of albite (*M* = 9%, SD = 12%), phlogopite 1 M mica (*M* = 4%, SD = 8%), clinochlore II2b (*M* = 3%, SD = 5%), and traces of microcline intermediate 1, magnetite, and pyrite. (Tables 1, 2 in supplementary material).

### XRF analysis

The chemical compositions of the CRPs are summarised in Tables 3, 4, 5 and 6 of Supplementary Material. The compositions of these largely lower Greenschist facies meta-silstones and meta-sandstones are dominated by SiO_2_ (*M* = 63%, SD = 8%), Al_2_O_3_ (*M* = 19%, SD = 5%), and Fe_2_O_3_ (*M* = 7%, SD = 2%). Notable traces included S (*M* = 1600 µg/g, SD = 3800 µg/g), Ba (*M* = 560 µg/g, SD = 250 µg/g), and As (*M* = 380 µg/g, SD = 650 µg/g).

### Particle size distribution

The analysis showed that samples were very similar in terms of their size distribution (Table 7 in supplementary material). The model fraction P2 (% 10 µm peak) was the most abundant fraction (*M* = 25.6, SD = 5). The largest fraction P1 (% 100 µm peak) was the second most abundant (*M* = 13.4, SD = 5.2). The nanoparticle fraction P3 (% < 1 µm) was only present in small amounts (*M* = 0.8, SD = 0.8). (Table 8 in supplementary material).

### Plasmid scission assay

**Fig. 1:**
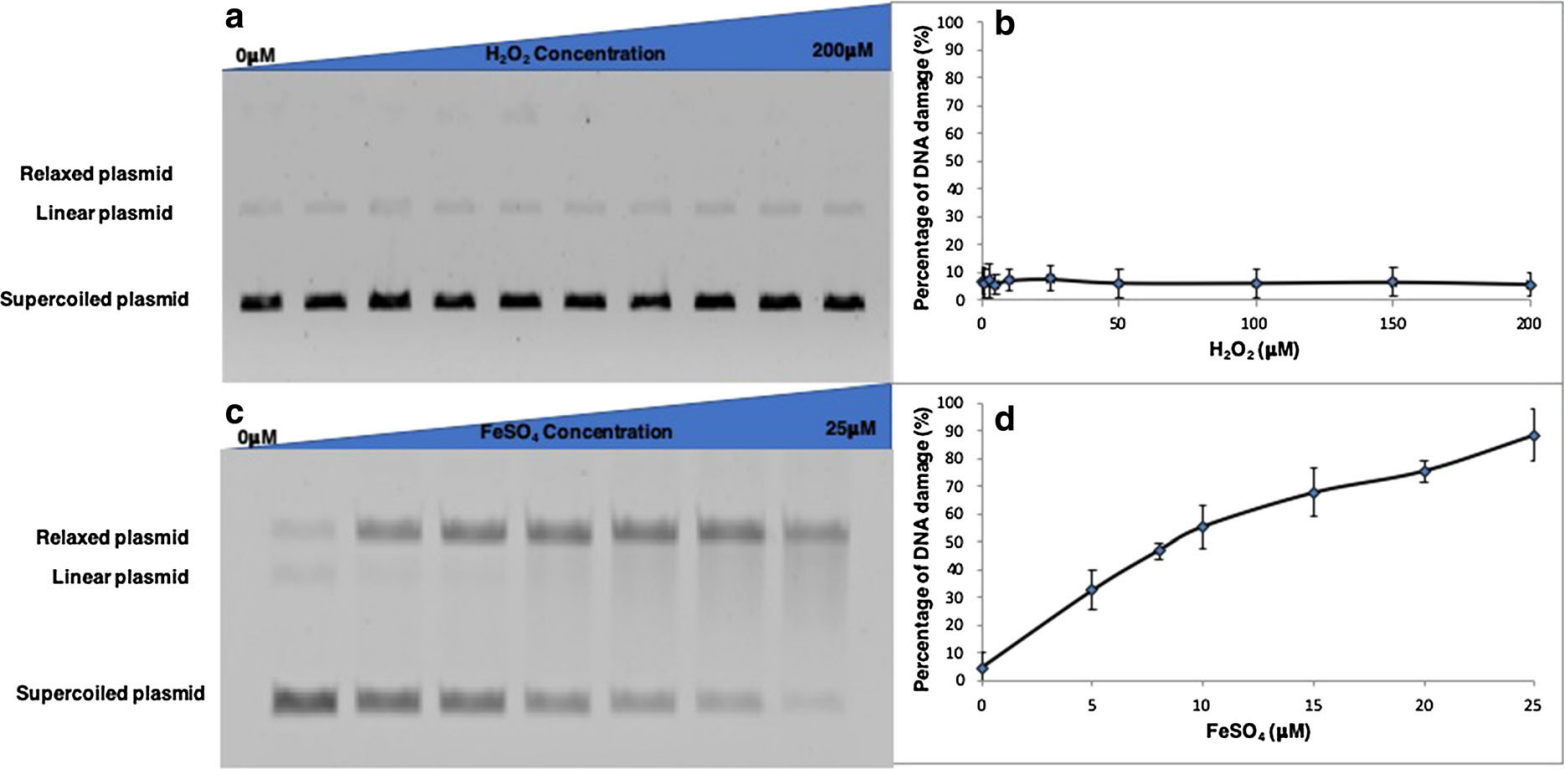
Production of DNA strand breaks by H_2_O_2_ and FeSO_4_. **a** electrophoresis results, **b**  %DNA damage induced by increasing amounts of H_2_O_2_)—pchAT plasmid DNA was incubated at 37 °C for 1 h with 0-200 μM H_2_O_2_ in elution buffer (10 mM Tris–HCl pH 8.5) (20 μl). **c** Electrophoresis results, **d**  % DNA damage induced by increasing amounts of FeSO_4_ in the presence of H_2_O_2_)—pchAT plasmid DNA was incubated at 37 °C for 1 h with 3.5 mM H_2_O_2_ and increasing concentration of FeSO_4_ (0–25 µM). Both reactions were stopped by the addition of loading buffer (Promega blue/orange 6 × loading dye). The samples were separated on 0.6% TBE-agarose gel at 90 V for 2 h. The different forms of plasmid were visualised on a Typhoon 9200 variable mode imager. The intensity of the different forms of plasmid (relaxed, linear, and supercoiled) in each lane was analysed using ImageQuantTM, and the level of damaged plasmid in each sample was calculated. Error bars represent the standard deviation (SD) of three independent experiments

Hydrogen peroxide (H_2_O_2_) alone did not induce substantial strand breaks, but in combination with increasing FeSO_4_ concentration, there was a dose-dependent increase in the proportion of damaged plasmid (Fig. 1). Negative controls (i.e. without particulates or FeSO_4_) gave rise to DNA damage of less than 10%, with this damage being independent of H_2_O_2_ concentration over the range 0–200 µM H_2_O_2_. These small levels of DNA damage are similar to those observed in earlier studies using this technique (Dumax-Vorzet et al. 2015), and we speculate reflect small amounts of damage caused by the DNA extraction procedure or during the incubation period.

**Fig. 2:**
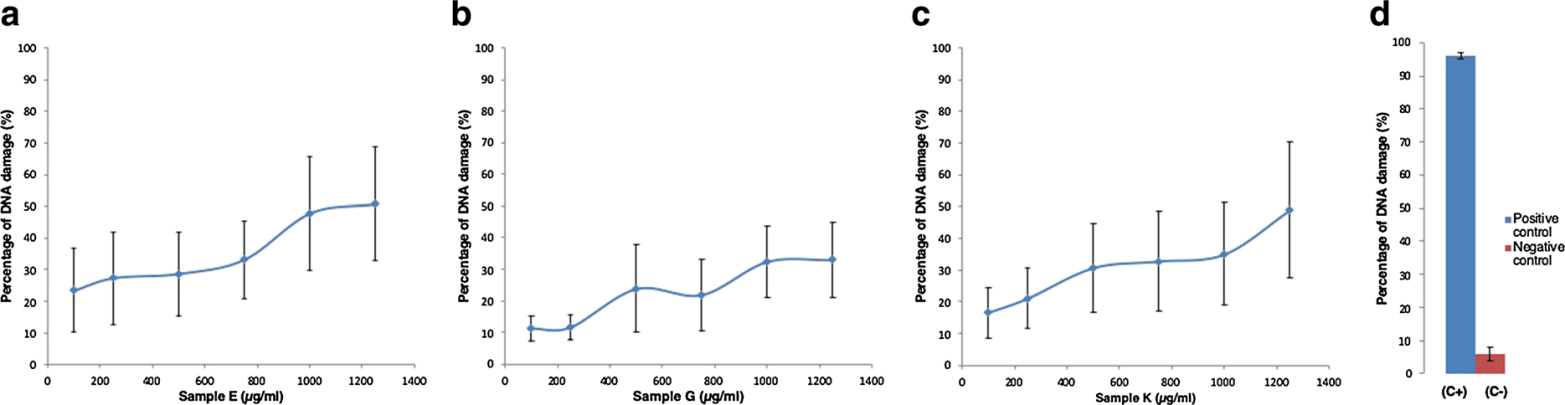
Production of DNA strand breaks by increasing amounts of CRPs (100–1250 µg/ml). **a**–**c** % DNA damage induced by increasing amounts of CRPs in the presence of H_2_O_2_ for three representative samples E, G, and K). Samples were suspended in distilled water (5 mg/ml) by sonication for a total of 3 min. Sonication was performed at 80% amplitude. The samples were used directly after being suspended without centrifugation. Plasmid DNA (5 ng) was incubated at 37 °C for 5 h with 3.5 mM H_2_O_2_ and increasing concentrations (100 μg/ml–1250 μg/ml) of the CRPs. (**d**  % DNA damage induced by negative and positive controls) positive [FeSO_4_ 25 µM, plasmid DNA (5 ng), H_2_O_2_ 3.5 mM, and elution buffer (10 mM Tris–HCl pH 8.5)] and negative [plasmid DNA (5 ng), H_2_O_2_ 3.5 mM, and elution buffer (10 mM Tris–HCl pH 8.5)] controls were also added. The reaction was stopped by the addition of loading buffer (Promega blue/orange 6 × loading dye). The samples were separated on 0.6% TBE-agarose gel at 100 V for 45 min. Three independents were carried out, and the mean for each sample was calculated. Error bars represent the standard deviation (SD) of three independent experiments

The addition of increasing amounts of CRPs to a reaction mix containing, pchAT plasmid DNA, H_2_O_2_, and elution buffer resulted in a dose-dependent increase in the proportion of damaged plasmid (Fig. 2).

**Fig. 3:**
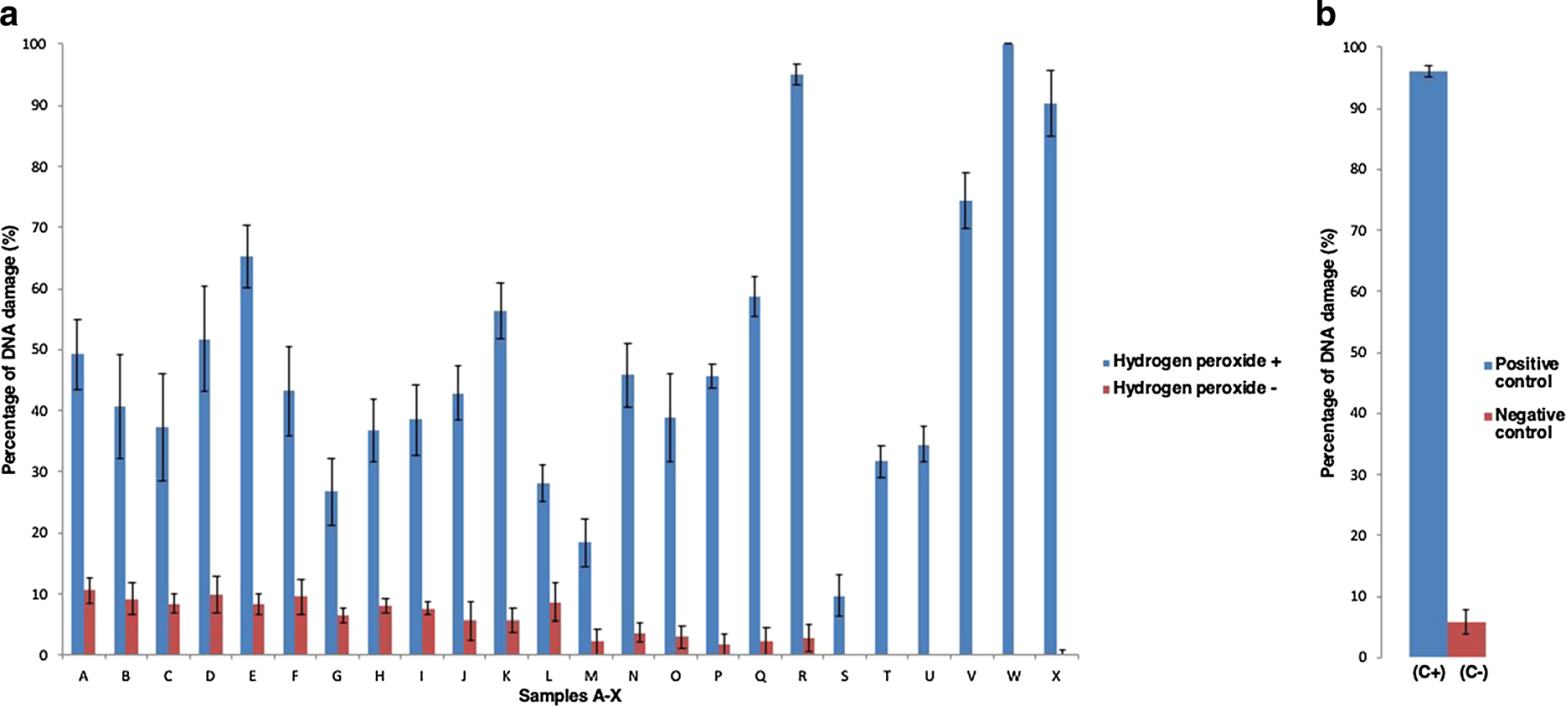
Production of DNA strand breaks by CRPs. (**a** % DNA damage induced by 1250 μg/ml of all CRPs in the presence of H_2_O_2_). Samples were suspended in distilled water (5 mg/ml) by sonication for a total of 3 min. Sonication was performed at 80% amplitude. The samples were used directly after being suspended without centrifugation. pchAT Plasmid DNA was incubated at 37 °C for 5 h with and without H_2_O_2_ at the highest concentration of the sample. (**b**  % DNA damage induced by negative and positive controls) positive [FeSO_4_ 25 µM, plasmid DNA (5 ng), H_2_O_2_ 3.5 mM, and elution buffer (10 mM Tris–HCl pH 8.5)] and negative [plasmid DNA (5 ng), H_2_O_2_ 3.5 mM, and elution buffer (10 mM Tris–HCl pH 8.5)] controls were also added. The reaction was stopped by the addition of loading buffer. The samples were separated on 0.6% TBE-agarose gel at 100 V for 45 min. Six independent experiments were carried out, and the mean for each sample was calculated. Error bars represent the standard deviation (SD) of six independent experiments

For samples incubated at the highest concentration of CRP (1250 µg/ml), the percentage of DNA strand breaks found in the absence of H_2_O_2_ was between 0 and 10%. However, when the samples were incubated with H_2_O_2_, the percentage of DNA strand breaks increased to between 10 and 100% depending upon the sample (Fig. 3). When comparing the percentage of DNA damage in the two groups (with and without H_2_O_2_), a significant difference was found for all samples using a paired samples *t* test (Table 9 in supplementary material). Also, when comparing the percentage of DNA damage within each group individually, a significant difference was found using a one-way analysis of variance (with H_2_O_2_ *p* < 0.001/without H_2_O_2_ *p* < 0.001).

**Fig. 4:**
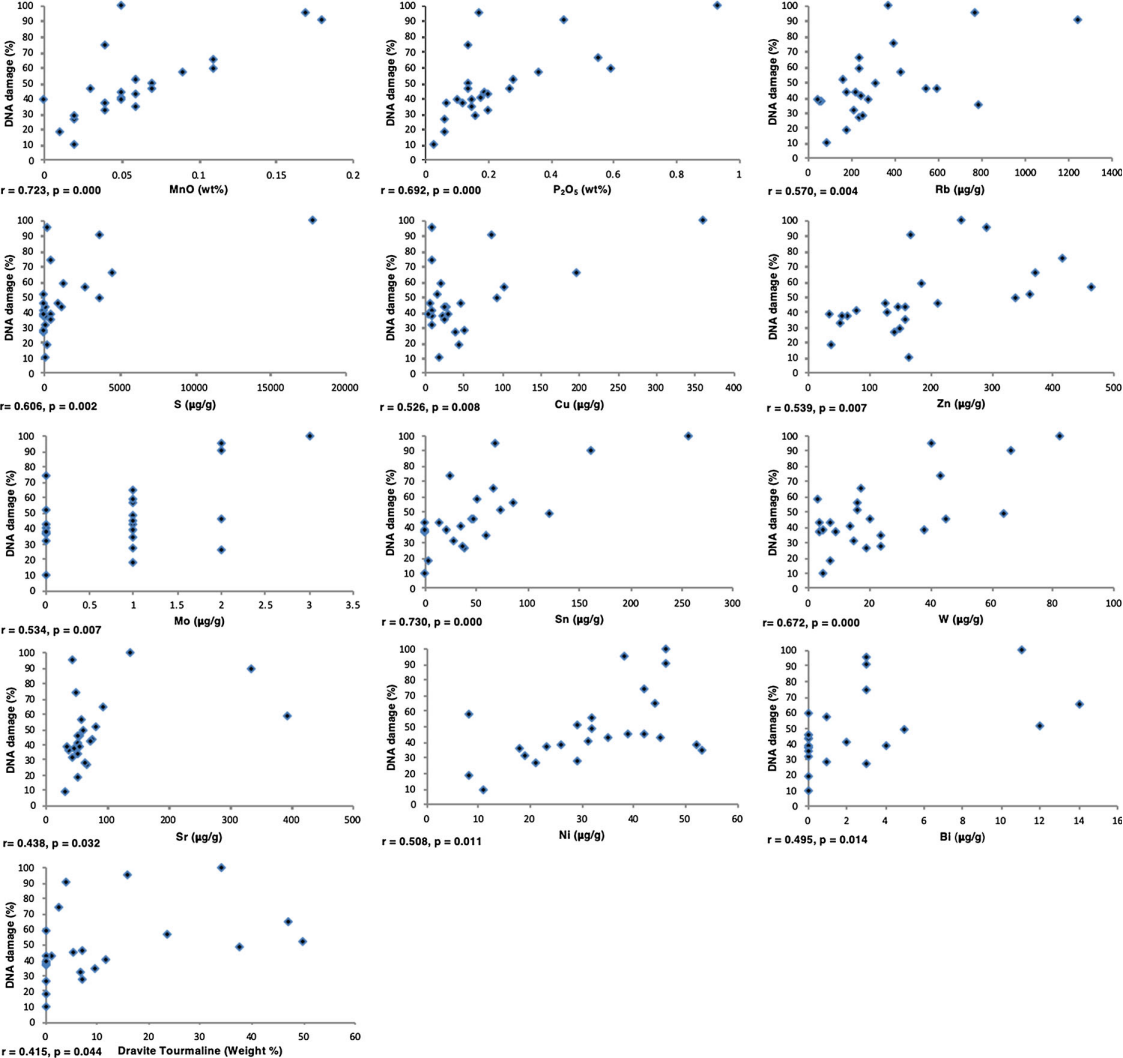
Correlation between significant components and DNA damage (*r* and *p* values shown were obtained from bivariate correlation analysis)

The percentage of plasmid DNA damage in the presence of H_2_O_2_ was correlated with MnO, P_2_O_5_, Rb, S, Cu, Zn, Mo, Sn, and W as well as Sn, Ni, Bi, and dravite tourmaline but to a lesser extent (Fig. 4). None of the particle size distribution parameters were correlated with the percentage of plasmid DNA damage. In a multiple linear regression model, MnO (*β* = 0.541, *p* < 0.000), S (*β* = 1.170, *p* < 0.002), Cu (*β* = − 0.761, *p* < 0.031), and Zn (*β* = 0.253, *p* < 0.035) were significant predictors of the percentage of DNA damage. These elements combined explained 83% of the variance (R^2^ = 0.834, *p* < 0.001). In the absence of H_2_O_2_, no correlation was found between the percentage of DNA damage and the physiochemical composition of the samples.

## Discussion

All samples collected from inside and around the Panasqueira mine area were able to induce DNA damage when incubated as a CRP with plasmid DNA in the presence of H_2_O_2_. The percentage of plasmid DNA damage varied significantly with MnO, S, Cu, and Zn, these chemical components also being significant predictors of DNA damage in a multivariate model. To the best of knowledge, this is the first study to determine direct apparent effects of MnO, S, Cu, and Zn in CRPs in a cell-free DNA scission assay, although it is noted that the large degree of covariance of these compositional parameters with other compositional parameters means that the DNA damage cannot be uniquely ascribed to each of these components and this represents a fundamental limitation of such toxicological studies involving real multicomponent geological materials. Previous studies have reported associations between these chemical components and DNA damage in cell-based studies (Alarifi et al. 2017; Frick et al. 2011; Hoffman et al. 2012; Linder 2012; Cervantes–Cervantes et al. 2005; Arciello et al. 2005; Zyba et al. 2016; Sharif et al. 2012; Ho and Ames 2002; Ho et al. 2003; Wysokinski et al. 2012). Manganese oxide nanoparticles have been associated with DNA strand breaks in human neuronal cells (Alarifi et al. 2017), and type-II alveolar epithelial cells (Frick et al. 2011). Sulphur (Hoffman et al. 2012) and copper (Linder 2012; Cervantes–Cervantes et al. 2005; Arciello et al. 2005) have been associated with superoxide and hydroxyl radicals which can result in oxidative stress and can lead to DNA damage. An increase in dietary zinc is actually known to reduce DNA damage (Zyba et al. 2016). Zinc deficiency on the other hand induces oxidative stress which leads to DNA damage (Sharif et al. 2012; Ho et al. 2003; Ho and Ames 2002). However, it has been reported before that zinc behaves differently in normal cells and cancer cells. Wysokinski et al. (2012) found that cancer cells exhibited higher levels of DNA damage in the presence of zinc, while in normal lymphocytes, such an effect was not found (Wysokinski et al. 2012). In this study, zinc levels were associated with an increase in DNA damage.

It has been previously reported that arsenic causes DNA strand breaks in mouse lungs (Yamanaka and Okada 1994), human fibroblasts (Mourón et al. 2006), and human HeLa S3 cells (Schwerdtle et al. 2003). However, in our study, we found no significant linear association between arsenic concentrations and DNA strand breaks, notwithstanding that the CRPs contained up to 3000 µg/g As. The lack of association between arsenic and DNA damage could have been attributed to its insolubility in a wide range of pH conditions (Flora 2014). Moreover, certain contaminants need to be converted from their original form by enzymes first in the human body to show any adverse effects. That especially applies for arsenic as its metabolism is a critical determinant of its toxic effects (Navas-Acien and Guallar 2008; Hughes et al. 2011; Jomova et al. 2011). This could be why arsenic was not found in this study to be associated with DNA damage as the assay was cell-free, while in the other previously mentioned studies, the assays were cell-based.

## Conclusion

Crushed rock powders of known chemical composition have been shown to induce variable levels of DNA damage in a cell-free assay. MnO, S, Cu, and Zn were significant predictors of this DNA damage. Further work is required to characterise the mechanism of DNA damage formation and to determine to what extent these cell-free studies correlate with cellular studies. In particular, the perhaps surprising lack of association of DNA damage with arsenic concentration in the crushed rock powders highlights how cell-free assays may not be representative of toxicity behaviour in human cells, but the assays nevertheless confirm that the toxicity of µm-scale particles may be strongly dependent upon their chemical and mineralogical composition.

## Appendix: Supplementary material

See Tables 1, 2, 3, 4, 5, 6, 7, 8, and 9.

**Table 1:** Mineralogical composition of crushed rock powders from Panasqueira; as determined by XRD

Sample	Mineral phase							
	Albite		Clinochlore IIb-2		Dravite tourmaline		Magnetite	
	Weight%	Error%	Weight%	Error%	Weight%	Error%	Weight%	Error%
A	1.0	0.2	0.8	0.3	37.7	0.8	ND	ND
B	16.4	0.8	ND	ND	11.8	0.7	ND	ND
C	15.8	0.7	2.6	0.6	ND	ND	ND	ND
D	0.6	0.2	1.5	0.4	49.8	0.9	ND	ND
E	ND	ND	ND	ND	47.0	0.9	ND	ND
F	26.3	1.6	17.6	1.2	1.4	0.8	ND	ND
G	ND	ND	3.1	1.0	ND	ND	ND	ND
H	8.0	0.5	5.9	0.4	ND	ND	ND	ND
I	11.4	0.4	3.3	0.3	ND	ND	ND	ND
J	24.6	0.9	8.7	0.5	ND	ND	ND	ND
K	ND	ND	ND	ND	23.4	0.7	ND	ND
L	ND	ND	1.1	0.9	7.3	1.0	ND	ND
M	ND	ND	2.2	0.6	ND	ND	ND	ND
N	0.4	0.9	0.6	0.8	7.0	1.0	ND	ND
O	15.9	0.9	7.8	0.7	ND	ND	ND	ND
P	8.9	1.0	ND	ND	5.4	0.9	ND	ND
Q	44.8	2.2	1.6	0.6	ND	ND	0.9	0.3
R	ND	ND	ND	ND	16.0	0.8	ND	ND
S	2.4	0.5	6.3	0.6	ND	ND	ND	ND
T	10.7	0.5	ND	ND	6.9	0.5	ND	ND
U	ND	ND	ND	ND	9.5	0.7	ND	ND
V	26.2	0.8	ND	ND	2.5	0.5	ND	ND
W	ND	ND	ND	ND	34.1	0.9	ND	ND
X	ND	ND	0.15	0.54	4.12	0.6	ND	ND

Sample	Mineral phase									
	Microcline intermediate 1		Muscovite 2M1		Phlogopite 1M Mica		Pyrite		Quartz	
	Weight %	Error %	Weight%	Error%	Weight%	Error%	Weight%	Error%	Weight%	Error%
A	2.4	0.5	19.4	0.7	ND	ND	ND	ND	38.9	0.8
B	ND	ND	17.3	1.0	ND	ND	ND	ND	54.5	1.3
C	3.4	0.8	18.9	0.9	ND	ND	ND	ND	59.3	1.2
D	2.4	0.6	10.1	0.6	ND	ND	ND	ND	35.6	0.8
E	ND	ND	20.4	0.7	ND	ND	ND	ND	ND	ND
F	ND	ND	54.8	1.9	ND	ND	ND	ND	32.7	0.8
G	ND	ND	85.1	1.2	ND	ND	ND	ND	11.8	0.8
H	2.6	0.6	17.7	0.8	ND	ND	ND	ND	65.7	1.0
I	2.6	0.4	12.5	0.5	ND	ND	ND	ND	70.1	0.7
J	ND	ND	34.9	1.1	ND	ND	ND	ND	31.8	1.0
K	2.6	0.5	22.2	0.8	ND	ND	ND	ND	51.7	0.9
L	ND	ND	48.0	1.6	ND	ND	ND	ND	43.6	1.6
M	ND	ND	53.5	1.5	ND	ND	ND	ND	44.3	1.5
N	ND	ND	37.4	1.6	12.2	0.8	ND	ND	42.5	1.6
O	ND	ND	41.7	1.3	ND	ND	ND	ND	34.7	1.2
P	ND	ND	32.1	1.5	20.6	1.1	ND	ND	33.1	1.4
Q	ND	ND	6.0	1.0	26.4	1.7	ND	ND	20.4	1.4
R	ND	ND	46.8	1.2	5.1	0.5	ND	ND	32.2	1.1
S	ND	ND	29.7	1.1	ND	ND	ND	ND	61.6	1.2
T	ND	ND	16.5	0.7	5.5	0.3	ND	ND	60.4	0.9
U	ND	ND	39.2	1.6	16.6	1.0	ND	ND	34.7	1.5
V	ND	ND	17.6	0.7	16.5	0.7	ND	ND	37.2	1.0
W	1.9	0.6	30.7	0.9	ND	ND	0.7	0.1	32.5	0.8
X	ND	ND	65.99	1.34	1.02	0.38	ND	ND	28.72	1.17

*ND not detected

**Table 2:** Descriptive statistics for abundance (wt%) of crystalline minerals identified by XRD in crushed rock powders from Panasqueira

Crystalline mineral	Minimum	Maximum	Mean	SD
Albite	ND	44.8	8.9	11.9
Clinochlore IIb2	ND	17.6	2.6	4.1
Dravite tourmaline	ND	49.8	11.0	15.6
Magnetite	ND	0.9	0.0	0.2
Microcline intermediate 1	ND	3.4	0.8	1.2
Muscovite 2M1	6.0	85.1	32.4	19.3
Phlogopite 1M Mica	ND	26.4	4.3	7.9
Pyrite	ND	0.7	0.0	0.2
Quartz	ND	70.1	39.9	16.8

**Table 3:** Chemical composition (major oxides) of crushed rock powders from Panasqueira; determined by XRF (Polya 1987, 1988)

Sample	Oxide (wt%)									
	Al_2_O_3_	CaO	Fe_2_O_3_	K_2_O	MgO	MnO	Na_2_O	P_2_O_5_	SiO_2_	TiO_2_
A	17.8	0.2	7.7	2.6	1.5	0.1	0.6	0.1	65.5	1.0
B	16.8	0.2	5.2	3.6	1.7	0.1	1.7	0.2	66.9	0.9
C	14.5	0.1	5.6	2.1	2.1	0.0	1.6	0.1	72.1	0.7
D	16.5	0.2	9.2	1.4	1.8	0.1	0.9	0.3	65.3	1.1
E	18.3	0.7	9.1	2.6	2.0	0.1	0.7	0.6	61.8	1.1
F	25.7	0.1	9.5	5.2	3.2	0.1	1.9	0.2	48.4	1.3
G	33.4	ND	5.4	7.4	1.3	0.0	0.8	0.1	46.1	1.9
H	13.9	ND	6.0	1.9	1.9	0.0	0.8	0.1	74.8	0.6
I	11.3	ND	3.2	1.3	1.3	ND	1.4	0.1	80.7	0.5
J	20.3	0.3	7.5	3.4	2.1	0.1	2.6	0.2	60.7	1.0
K	15.0	0.4	5.6	3.1	1.5	0.1	0.4	0.4	71.3	0.8
L	20.3	0.0	8.2	4.1	1.7	0.0	0.5	0.2	58.7	1.1
M	24.4	0.0	5.9	4.3	0.9	0.0	0.8	0.1	60.0	1.0
N	20.0	0.2	5.6	5.0	2.1	0.0	1.2	0.1	63.7	1.0
O	21.1	0.2	7.5	4.1	2.0	0.1	1.6	0.2	60.9	1.0
P	18.7	0.3	7.8	5.5	2.5	0.1	1.5	0.3	59.7	1.0
Q	17.3	3.7	7.6	3.2	4.4	0.1	3.2	0.6	55.3	1.2
R	20.4	0.2	8.3	6.0	2.5	0.2	0.4	0.2	57.4	1.1
S	19.3	ND	5.1	2.9	2.1	0.0	0.2	0.0	65.9	0.8
T	14.4	0.3	4.2	3.0	1.6	0.0	1.5	0.2	73.2	0.6
U	20.5	0.2	7.0	5.7	2.2	0.1	1.0	0.2	62.3	0.9
V	16.0	0.3	6.2	3.5	2.2	0.0	3.0	0.1	66.6	0.9
W	19.2	1.1	7.1	3.4	2.1	0.1	0.5	0.9	63.3	1.0
X	21.4	0.6	6.5	7.0	2.2	0.2	0.1	0.4	57.4	0.9

*ND* not detected; 0.0 indicates < 0.05

**Table 4:** Chemical composition (trace elements) of crushed rock powders from Panasqueira; as determined by XRF (Polya 1987, 1988)

Sample	Element (µg/g)																							
	As	Ba	Bi	Ce	Co	Cr	Cu	La	Mo	Nb	Ni	Pb	Rb	S	Sb	Sn	Sr	Th	U	V	W	Y	Zn	Zr
A	3164	274	5	150	13	120	94	32	1	13	32	10	308	3733	3	122	58	8	3	116	64	31	338	190
B	113	564	2	48	11	99	8	30	ND	12	31	4	242	35	ND	35	51	10	2	96	14	33	78	258
C	10	402	ND	45	14	83	9	20	ND	11	23	9	62	49	ND	ND	44	7	3	76	4	23	63	177
D	730	419	12	99	7	130	17	42	ND	9	29	6	162	9	ND	73	80	12	3	134	16	31	360	193
E	883	365	14	100	15	135	197	35	1	14	44	7	237	4570	ND	66	90	13	4	141	17	40	368	189
F	99	928	ND	92	10	151	27	58	1	18	35	12	179	64	ND	ND	74	13	3	171	4	41	144	264
G	235	1330	3	125	10	218	40	62	2	26	21	130	236	40	1	38	64	20	5	222	19	64	140	496
H	94	354	ND	32	8	73	23	24	ND	8	18	37	56	61	ND	ND	36	7	ND	67	9	25	55	175
I	3	214	ND	37	10	66	4	17	ND	8	26	ND	43	25	ND	ND	33	3	3	59	5	30	33	178
J	330	633	ND	85	18	129	25	34	ND	14	45	3	217	1140	ND	14	72	11	2	141	7	37	158	193
K	550	257	1	63	11	89	104	25	1	10	32	55	426	2732	ND	87	57	7	2	78	16	27	460	221
L	417	608	1	69	13	127	51	38	1	14	29	23	250	28	ND	37	61	11	4	129	24	28	147	199
M	10	848	ND	68	7	136	44	44	1	16	8	33	175	201	ND	4	51	11	3	156	7	40	35	228
N	60	646	ND	68	16	116	46	43	2	14	42	18	546	956	4	46	54	5	3	122	45	42	209	248
O	44	688	4	71	12	130	29	38	1	15	52	4	273	487	ND	21	53	11	3	141	38	37	126	189
P	206	701	ND	61	8	121	6	37	1	13	39	5	589	32	ND	47	50	4	3	135	20	46	125	200
Q	17	596	ND	68	17	37	20	39	1	9	8	13	236	1362	ND	51	391	6	2	109	3	28	182	227
R	553	561	3	85	8	130	8	35	2	15	38	ND	763	249	ND	69	40	8	4	141	40	45	288	201
S	14	606	ND	64	10	88	18	37	ND	12	11	13	82	67	ND	0	31	9	2	92	5	27	164	196
T	137	442	ND	42	6	84	8	21	ND	9	19	4	214	83	ND	28	40	5	3	70	15	23	50	228
U	199	689	ND	65	15	124	26	36	1	13	53	3	785	515	ND	60	51	9	4	134	24	43	157	195
V	159	362	3	69	13	109	9	34	ND	13	42	35	396	459	ND	25	47	7	3	102	43	36	414	187
W	656	470	11	110	19	137	362	34	3	13	46	98	370	17,822	5	257	135	9	4	145	82	52	248	184
X	462	550	3	64	10	116	86	43	2	22	46	16	1241	3764	6	162	333	9	4	130	66	46	166	174

*ND* not detected

**Table 5:** Descriptive statistics for chemical composition (major oxides) of Panasqueira crushed rock powders (*n* = 24) determined by XRF

Oxide	Minimum/maximum (wt%)	Mean/SD (wt%)
Al_2_O_3_	11.3/33.4	19.0/4.5
CaO	0.0/3.7	0.4/0.8
Fe_2_O_3_	3.2/9.5	6.7/1.6
K_2_O	1.3/7.4	3.8/1.7
MgO	0.9/4.4	2.0/0.7
MnO	0.0/0.2	0.1/0.0
Na_2_O	0.1/3.2	1.2/0.8
P_2_O_5_	0.0/0.9	0.2/0.2
SiO_2_	46.1/80.7	63.2/7.9
TiO_2_	0.5/1.9	1.0/0.3

**Table 6:** Descriptive statistics for chemical composition (trace elements) of Panasqueira crushed rock powders (*n* = 24) determined by XRF

Element	Minimum/maximum (µg/g)	Mean/SD (µg/g)
As	3/3164	381/646
Ba	214/1330	563/244
Bi	0/14	3/5
Ce	32/150	74/28
Co	6/19	12/4
Cr	37/218	115/36
Cu	4/362	53/80
La	17/62	36/11
Mo	0/3	1/1
Nb	8/26	13/5
Ni	8/53	32/14
Pb	0/130	22/32
Rb	43/1241	337/279
S	9/17,822	1604/3714
Sb	0/6	1/2
Sn	0/257	52/60
Sr	31/391	83/89
Th	3/20	9/4
U	0/5	3/1
V	59/222	121/38
W	3/82	25/22
Y	23/64	37/10
Zn	33/460	188/124
Zr	174/496	216/65

Zero used as default to indicate below detection limit

**Table 7:** Particle size distribution of crushed rock powders from Panasqueira; as determined by Beckman Coulter LS 13 320 Particle Sizing Analyser

Sample	Particle size parameters				
	D10 (µm)	D50 (µm)	P1 (% 100 µm peak)	P2 (% 10 µm peak)	P3 (% < 1 µm)
A	3.5	27.7	16.1	16.7	1.1
B	3.6	15.0	12.0	25.0	0.3
C	3.4	13.2	16.6	27.1	0.1
D	2.8	17.5	16.5	19.8	1.9
E	2.9	14.1	11.7	23.3	1.1
F	3.3	12.4	15.4	27.7	0.0
G	3.0	10.2	11.8	29.8	0.0
H	2.7	11.9	16.8	25.6	0.6
I	2.2	9.3	15.4	25.2	1.6
J	2.2	7.6	4.4	31.3	1.0
K	2.9	15.3	9.5	22.1	1.2
L	3.1	9.9	6.8	34.8	0.1
M	2.7	8.6	8.7	34.0	0.2
N	2.8	11.1	13.1	26.6	0.3
O	2.6	9.4	11.5	29.5	0.3
P	4.5	20.6	14.0	20.0	0.3
Q	2.2	21.9	22.1	15.5	3.2
R	3.5	15.7	8.9	23.5	0.5
S	2.6	9.6	15.3	26.4	0.2
T	3.8	21.5	19.1	19.3	0.5
U	2.7	9.7	11.8	30.1	0.1
V	2.0	8.5	5.8	28.0	2.8
W	2.4	9.6	12.7	24.9	0.6
X	3.0	12.0	14.8	27.5	0.5

D10 is the diameter at which 10% of a sample’s mass is comprised of smaller particles. D50 is the diameter at which 50% of a sample’s mass is comprised of smaller particles. P1 (% 100 µm peak) describes the largest fraction peak (100 µm). P2 (% 10 µm peak) describes the modal fraction peak (10 µm). *P3 (% < 1 µm) describes the sub-micron particle fraction peak (< 1 µm)

**Table 8:** Descriptive statistics for particle size distribution parameters for crushed rock powders from Panasqueira

Particle size parameter	Minimum/maximum	Mean/SD
D10 (µm)	2.0/4.5	2.9/0.6
D50 (µm)	7.6/27.7	13.4/5.2
P1 (% 100 µm peak)	4.4/22.1	12.9/4.2
P2 (% 10 µm peak)	15.5/34.8	25.6/5.0
P3 (% < 1 µm)	0.0/3.2	0.8/0.8

**Table 9:** A comparison of the level of DNA strand breaks (mean/standard deviation of six independent experiments) between powdered rock samples from Panasqueira (*n* = 24) incubated with and without H_2_O_2_ using a paired samples *t* test

Sample	With H_2_O_2_	Without H_2_O_2_	Ratio for with/without H_2_O_2_
	Mean/SD	Mean/SD	
A	49.18/5.82	10.51/2.04	4.7*
B	40.67/8.54	9.17/2.53	4.4*
C	37.20/8.77	8.33/1.59	4.5*
D	51.73/8.56	9.88/2.94	5.2*
E	65.31/5.12	8.20/1.71	8.0*
F	43.19/7.29	9.47/2.96	4.6*
G	26.71/5.41	6.51/1.16	4.1*
H	36.74/5.05	8.02/1.22	4.6*
I	38.54/5.74	7.61/1.12	5.1*
J	42.82/4.41	5.56/3.13	7.7*
K	56.37/4.49	5.65/1.92	10.0*
L	28.16/2.91	8.67/3.01	3.2*
M	18.37/3.94	2.16/2.13	8.5*
N	45.79/5.26	3.70/1.65	12.4*
O	38.80/7.18	3.00/1.80	12.9*
P	45.60/1.88	1.70/1.77	26.8*
Q	58.72/3.20	2.24/2.17	26.2*
R	95.06/1.74	2.71/2.24	35.1*
S	9.71/3.45	0.00/0.00	*−
T	31.71/2.58	0.00/0.00	*−
U	34.49/2.83	0.00/0.00	*−
V	74.37/4.65	0.00/0.00	*−
W	100.00/0.00	0.02/0.04	5567*
X	90.32/5.24	0.28/0.63	323*

*There was a significant difference for all samples when incubated with and without H_2_O_2_. All *p* values for the *t* test were < 0.001

*(−) values could not be calculated
